# Population Pharmacokinetics of Artemether, Dihydroartemisinin, and Lumefantrine in Rwandese Pregnant Women Treated for Uncomplicated Plasmodium falciparum Malaria

**DOI:** 10.1128/AAC.00518-18

**Published:** 2018-09-24

**Authors:** Jesmin Lohy Das, Stephen Rulisa, Peter J. de Vries, Petra F. Mens, Nadine Kaligirwa, Steven Agaba, Joel Tarning, Mats O. Karlsson, Thomas P. C. Dorlo

**Affiliations:** a Department of Pharmaceutical Biosciences, Uppsala University, Uppsala, Sweden; b Department of Clinical Research, University of Kigali, Kigali, Rwanda; c Tergooi Hospital, Hilversum, the Netherlands; d Department of Medical Microbiology, Academic Medical Center, Amsterdam, the Netherlands; e Center for Treatment and Research on AIDS, Malaria and TB (TRAC PLUS), Rwanda Biomedical Center, Kigali, Rwanda; f Mahidol-Oxford Tropical Medicine Research Unit, Faculty of Tropical Medicine, Mahidol University, Bangkok, Thailand; g Centre for Tropical Medicine and Global Health, Nuffield Department of Medicine, University of Oxford, Oxford, United Kingdom; h Department of Pharmacy & Pharmacology, Antoni van Leeuwenhoek Hospital/the Netherlands Cancer Institute, Amsterdam, the Netherlands

**Keywords:** artemether, dihydroartemisinin, lumefantrine, malaria, pharmacokinetics, pharmacometrics, pregnancy

## Abstract

The artemisinin-based combination therapy artemether-lumefantrine is commonly used in pregnant malaria patients. However, the effect of pregnancy-related changes on exposure is unclear, and pregnancy has been associated with decreased efficacy in previous studies. This study aimed to characterize the population pharmacokinetics of artemether, its active metabolite dihydroartemisinin, and lumefantrine in 22 Rwandese pregnant women in their second (*n* = 11) or third (*n* = 11) trimester with uncomplicated Plasmodium falciparum malaria. These patients were enrolled from Rwamagana district hospital and received the standard fixed oral dose combination of 80 mg of artemether and 480 mg of lumefantrine twice daily for 3 days. Venous plasma concentrations were quantified for all three analytes using liquid chromatography coupled with tandem mass spectroscopy, and data were analyzed using nonlinear mixed-effects modeling. Lumefantrine pharmacokinetics was described by a flexible but highly variable absorption, with a mean absorption time of 4.04 h, followed by a biphasic disposition model. The median area under the concentration-time curve from 0 h to infinity (AUC_0–∞_) for lumefantrine was 641 h · mg/liter. Model-based simulations indicated that 11.7% of the study population did not attain the target day 7 plasma concentration (280 ng/ml), a threshold associated with increased risk of recrudescence. The pharmacokinetics of artemether was time dependent, and the autoinduction of its clearance was described using an enzyme turnover model. The turnover half-life was predicted to be 30.4 h. The typical oral clearance, which started at 467 liters/h, increased 1.43-fold at the end of treatment. Simulations suggested that lumefantrine pharmacokinetic target attainment appeared to be reassuring in Rwandese pregnant women, particularly compared to target attainment in Southeast Asia. Larger cohorts will be required to confirm this finding.

## Abstract (precis)

The artemisinin-based combination therapy artemether-lumefantrine is commonly used in pregnant malaria patients. However, the effect of pregnancy-related changes on exposure is unclear, and pregnancy has been associated with decreased efficacy in previous studies.

## INTRODUCTION

Despite recent remarkable progress in the battle against malaria, this parasitic disease continues to have a devastating impact on public health (1). Pregnant women in particular are susceptible to malaria, and it is estimated that 125 million women are at risk of malaria every year (2). Pregnant women with malaria may appear asymptomatic and are at greater risk to develop severe Plasmodium falciparum malaria in low-transmission settings (2), and malaria during pregnancy has been associated with maternal mortality, risk of abortion, stillbirth, neonatal morbidity, and low birth weight (3).

The oral fixed-dose combination of artemether (ARM) and lumefantrine (LF) given as 80 mg and 480 mg twice daily for 3 days is recommended and widely used in pregnant women, particularly in the second and third trimesters (1, 4). ARM and LF work in a complementary fashion against Plasmodium falciparum: the short-acting ARM and its metabolite dihydroartemisinin (DHA) lead to rapid elimination of malarial biomass, while the long-acting LF eliminates the remaining residual parasites to prevent recrudescence.

The dynamic and profound physiological changes during pregnancy have been reported to influence the pharmacokinetics (PK) of various drugs, including antimalarials (5). The changes, which include gestational weight gain, plasma protein binding decrease, lipid concentration increase, and increases or decreases in activities of multiple cytochrome P450 enzymes (such as CYP3A4, CYP2C9, and CYP2A6), are often associated with lower drug concentrations, possibly leading to lower antimalarial cure rates in pregnant women with malaria infection (6–8). Nevertheless, these changes are often not taken into account in the dosing regimens during pregnancy.

The LF plasma concentration on day 7 is considered the most important PK target for LF to attain in malaria (9–11). Two different venous LF day 7 plasma target concentrations have been reported in the literature, i.e., 280 and 175 ng/ml (9, 12). A large pooled analysis showed that day 7 concentrations of ≥200 ng/ml were associated with a cure rate above 98%; hence, 280 ng/ml was used in the present study as the target (11). In addition, the compromised host immunity during pregnancy probably necessitates this relatively higher PK target (11).

LF exposure in pregnant women compared to nonpregnant women has been reported to be lower in Uganda and the borders of northwestern Thailand, where more than 30% of patients had day 7 LF concentrations below 280 ng/ml (13, 14). The low day 7 LF concentrations were associated with a low cure rate in Thailand but not in Uganda (14, 15). This was later considered an argument to consider extending the dosing regimen from 3 days to 5 days for pregnant women (13). The reduction of LF exposure in pregnancy and its impact on clinical outcome are, however, unclear and appear to be population specific; therefore, the suggested dose extension is still open for debate. Similarly, lower drug exposure has been reported for ARM-DHA in pregnant women (16). ARM is a lipid-soluble derivative of artemisinin and has been reported to exhibit nonlinear time-dependent PK caused by autoinduction of its clearance (17–20). Studies characterizing the PK of ARM in pregnant women have been conducted previously (7, 21); however, these studies mostly used noncompartmental analysis. Nevertheless, autoinduction was not investigated with compartmental analysis mainly because observations were made only after the last dose of the treatment (21).

The aim of this study was to characterize the population PK properties of LF, ARM, and its active metabolite DHA in pregnant women in the second and third trimesters presenting with uncomplicated Plasmodium falciparum malaria in Rwanda. The developed model for LF was used to evaluate LF day 7 concentrations for the standard dosing regimen (3 days) and the need to adopt the suggested alternative dosing regimen of 5 days in this particular population. ARM has been described to exhibit autoinduction of clearance, and the appropriateness of this phenomenon was evaluated in this analysis (4, 17). Since LF and ARM were administered as a fixed-dose combination, correlation between absorption rate and extent of both drugs was explored by modeling both drugs simultaneously in an attempt to improve individual model estimates.

## RESULTS

### Study demographics and data.

Twenty-two Rwandese pregnant patients in their second (range for estimated gestational age [EGA], 15.7 to 27.6 weeks) or third trimester (range for EGA, 28.3 to 39.0 weeks) with a median EGA of 27.9 weeks were studied, and their blood plasma samples were analyzed for both LF and ARM-DHA. The treatment was well tolerated. The demographics of these patients are shown in Table 1.

**TABLE 1:** Baseline study demographics of patients

Parameter	Median value (range)		Total
	2nd trimester	3rd trimester	
No.	11	11	22
Age (yr)	24 (19–39)	26 (18–29)	26 (18–39)
Baseline parasitemia (parasites/μl)	34,800	22,825	24,970
	(11,700–96,000)	(3,060–160,000)	(3,060–160,000)
Body wt (kg)	59.0 (40.0–65.0)	59.0 (45.0–65.0)	59.0 (40.0–65.0)
Body mass index	21.8 (15.6–25.2)	22.0 (18.2–25.4)	21.9 (15.6–25.4)
Estimated gestational age (wks)	24.6 (15.7–27.6)	32.6 (28.3–39.0)	27.9 (15.7–39.0)
Temp (°C)	36.5 (35.1–38.6)	37.0 (34.9–38.4)	36.8 (34.9–38.6)

### Samples and data.

A total of 363 blood plasma samples for LF and 387 blood plasma samples for ARM and DHA were analyzed. Nine samples out of these were excluded for LF, ARM, and DHA due to hemolysis. For LF less than 8% of the data were below the lower limit of quantification (BLLOQ), while for ARM and DHA 24% and 28%, respectively, were.

### Population pharmacokinetics of lumefantrine.

**FIG 1:**
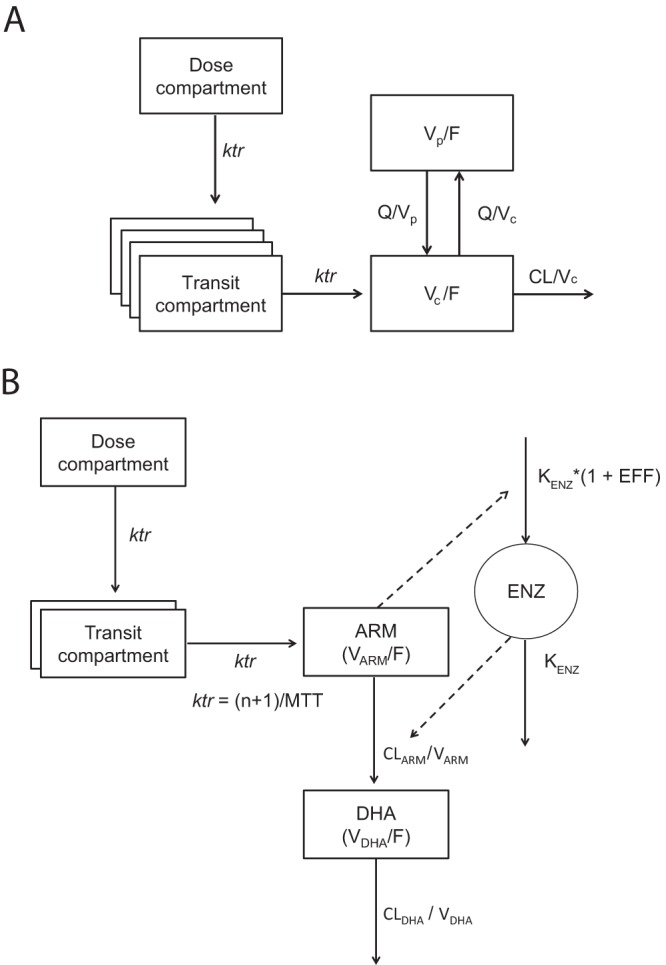
(A) Schematic of the structural population pharmacokinetic model for lumefantrine (LF). CL/F, central elimination clearance of LF; Q/F, intercompartmental clearance; *ktr*, transit absorption rate constant [*ktr* = (*n* + 1)/mean transit time]; *V_c_*, apparent volume of distribution of central compartment; *V_p_*, apparent volume of distribution of peripheral compartment. (B) Schematic of the structural artemether (ARM) and dihydroartemisinin (DHA) model. CL_ARM_, elimination clearance of ARM; CL_DHA_, elimination clearance of DHA; *V*_ARM_, apparent volume of distribution of ARM of central compartment; *V*_DHA_, apparent volume of distribution of DHA of central compartment. The enzyme model is linked to the drug model by ARM concentrations which stimulate the enzyme production rate (*K*_ENZ_). This increases the enzyme amount (ENZ) following an *E*_max_ model relation which, in turn, increases ARM's clearance (CL_ARM_).

The final structural model for LF (Fig. 1) was a two-compartment disposition model with an absorption model consisting of 5 first-order transit compartments delivering the absorbed amount to the central compartment, with mean absorption time (MAT) of 4.04 h and terminal elimination half-life of ∼4 days (Table 2). This was consistent with previous studies reporting population PK of LF (15, 22).

**TABLE 2:** Parameter estimates from the final population pharmacokinetic model^*a*^

Parameter (fixed effects)	Population estimate (% RSE)	90% CI	BSV/BOV, % CV (% RSE)	90% CI
Lumefantrine				
F	1 *fixed*		144 (19.7)^*b*^	106 to 189^*b*^
Box-Cox shape parameter for BSV on F	−0.605 (34.9)	−0.590 to −0.180		
MTT (h)	4.04 (5.16)	3.71 to 4.41	132 (37.9)	72.6 to 178
			46.0 (43.6)^*b*^	13.1 to 64.5^*b*^
CL/F (liters/h)	4.49 (6.59)	4.18 to 5.17		
*V_c_*/F (liters)	139 (6.77)	119 to 149	48.7 (56.8)	17.8 to 77.8
Q/F (liters/h)	0.924 (13.3)	0.770 to 1.21		
*V_p_*/F (liters)	111 (8.69)	96.5 to 129		
RUV (%)	48.7 (4.82)	45.8 to 53.5		
Artemether				
F	1 *fixed*		57.6 (36.8)	43.2 to 78.8
			48.2 (35.6)^*b*^	39.1 to 63.9^*b*^
MTT (h)	0.738 (12.5)	0.569 to 0.840	110 (32.5)	86.2 to 143.2
			53.2 (21.6)^*b*^	54.1 to 97.9^*b*^
CL_ARM_/F (liters/h)	467 (17.9)	298 to 508	27.9 (44.1)	21.5 to 43.5
*V*_ARM_/F (liters)	3,000 (14.1)	2,050 to 3,180	20.5 (43.1)	15.4 to 31.0
RUV (%)	98.4 (5.54)	92.0 to 108		
*E*_max_ (h^−1^)	0.986 (22.8)	0.623 to 1.42		
EC_50_ (nM)	9.37 (25.4)	6.16 to 14.4		
TIME_ENZ_ (h)	30.4 (42.1)	7.59 to 41.9		
Dihydroartemisinin				
CL_DHA_/F (liters/h)	611 (15.4)	486 to 782	20.7 (50.2)	12.9 to 29.9
*V*_DHA_/F (liters)	137 (38.9)	99.8 to 251	40.5 (48.9)	17.5 to 51.5
RUV (%)	113 (6.01)	109 to 129		

a Coefficient of variation (CV) for BSV and BOV was calculated as 100 × (variance)^1/2^. Relative standard errors (RSE) were calculated as 100 × (standard deviation/mean). The 90% confidence intervals (CI) of parameter estimates were obtained with the sampling importance resampling (SIR) routine. ARM, artemether; BOV, between-occasion variability; BSV, between-subject variability; CL, clearance; DHA, dihydroartemisinin; *E*_max_, maximum effect of autoinduction; EC_50_, artemether concentration for which the autoinduction effect is half of the maximum effect; F, relative bioavailability; MTT, mean transit time; *V_c_*, volume of distribution of central compartment of lumefantrine; Q, clearance of peripheral compartment; RUV, residual unexplained variability; *V_p_*, volume of distribution of peripheral compartment of lumefantrine; *V*, volume of distribution; RUV, residual unexplained variability; TIME_ENZ,_ half-life of the autoinduced enzyme.

b Values indicate BOV; all others are BSV.

**FIG 2:**
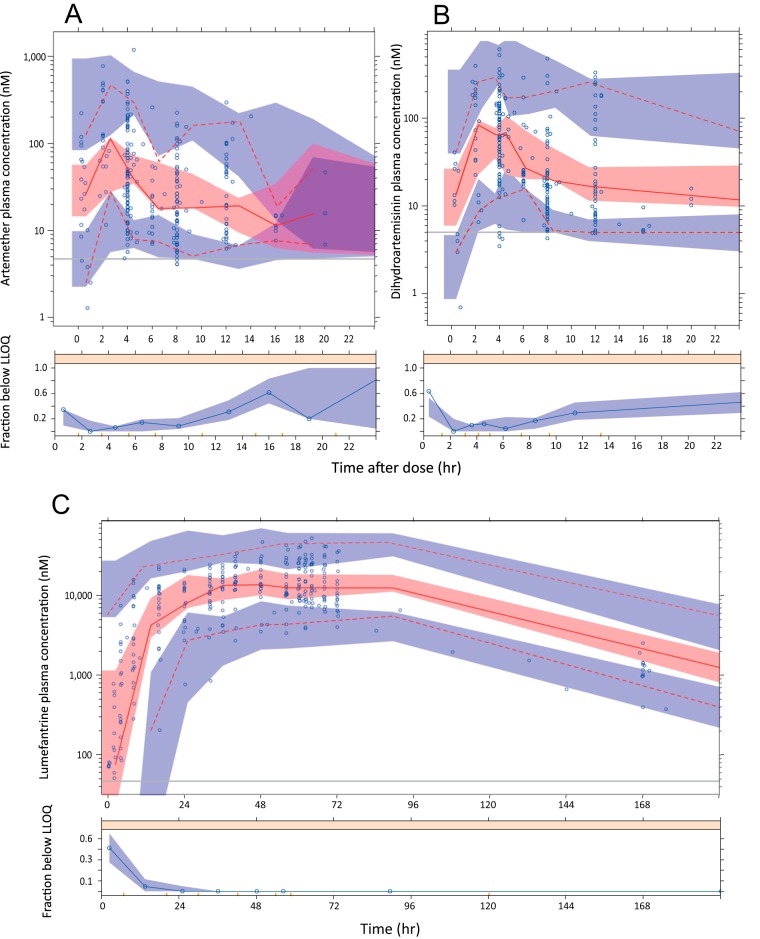
Prediction-corrected visual predictive checks (pcVPC) of the parent, artemether (A), and its active metabolite dihydroartemisinin (B) and visual predictive check for lumefantrine (C). The median (red solid line) and 5th and 95th percentiles (red dashed lines) of the observed data (blue circles) are compared to the 95% confidence intervals (shaded areas) for the respective percentiles of the simulated data (*n* = 1,000). The horizontal gray line represents the lower limit of quantification (LLOQ). For pcVPC, correction was performed after stratification and binning. The lower panel shows the fraction of observed data below the LLOQ (open circles) overlaid with the 95% prediction interval of the fraction of simulated data below the LLOQ (shaded area).

A frequentist prior approach applied to fixed PK parameters of LF (i.e., clearance, volume of distribution, and absorption parameter) allowed previously reported PK information to support aspects that could not be characterized by the current study data alone, i.e., implementation of a peripheral compartment. The BLLOQ data were not included for LF in this analysis since the proportion was less than 10%. There was also no bias in BLLOQ data observed in the visual predictive checks (VPC) (Fig. 2) when these data were treated as missing or categorical data. Between-subject variability (BSV) was estimated for all structural parameters except for apparent clearance (CL/F), intercompartmental clearance (Q/F), and the volume of distribution of the peripheral compartment (*V_p_*/F) of LF, which were estimated with poor precision (>50% residual standard error [RSE]). The final PK model structure for LF is depicted in Fig. 1A. The final PK parameter estimates are summarized in Table 2.

The addition of between-occasion variability (BOV) was significant for mean transit time (MTT) (change in objective function value [ΔOFV] = −15.1) and relative bioavailability (F) (ΔOFV = −124.2). The description of the outer data percentiles and hence model fit was improved when Box-Cox transformation of the distribution of the BSV for F was applied (ΔOFV = −120.85). Eta shrinkages for BSV computed for the final LF parameter estimates were 25.8% for *V_p_*/F and 11.1% for MTT. Eta shrinkage for BOV ranged between 14.5% and 39.4% for MTT and 32.5% to 65.3% for F. Computed epsilon shrinkage (residual variability) for LF was 19.9%. The median area under the plasma concentration-time curve from 0 h to infinity (AUC_0–∞_) for LF was 641 h · mg/liter (95% confidence interval [CI], 596 to 858 h · mg/liter). The predictive performance of the LF model is depicted in the VPC in Fig. 2.

### Population pharmacokinetics of artemether and its metabolite dihydroartemisinin.

The Laplacian estimation method was used to estimate model parameters for ARM-DHA. The final ARM-DHA model consisted of one-compartment disposition models for both ARM and DHA, with the assumption of irreversible *in vivo* conversion of ARM to DHA (Fig. 1B). An absorption model consisting of 2 transit compartments was superior to other explored absorption models.

From the observed data, the peak ARM concentration 2 h after the first dose was approximately 3 times (median 3.75) higher than that 2 h after the last dose, while this ratio was 0.87 for DHA. This suggested the possibility of a time-dependent decrease in ARM concentrations, i.e., enzyme induction of its own clearance, as previously described (17, 20, 23). The autoinduction effect (EFF) of the ARM concentration on the half-life of the susceptible enzymes, believed to represent CYP3A4, was modeled using a simple nonlinear relationship as described below (equation 1):

(1) $$\text{EFF}=\frac{E_{\text{max}}\times \text{CP}}{{EC}_{50}+\text{CP}}$$

where *E*_max_ represents the maximal autoinduction effect, EC_50_ represents the ARM concentration when the autoinduction is half maximal, and CP is the ARM plasma concentration.

The estimated enzymatic half-life was 30.4 h, while the EC_50_ was 9.37 nM. Typical clearance of ARM increased 43% at occasion six compared to occasion one.

Approaches to describe autoinduction of the clearance using alternative physiologically plausible models were also explored during the model building, such as (i) ARM being cleared via two elimination pathways, i.e., inducible and noninducible clearances (both estimated), where the inducible pathway produces DHA, and (ii) similar to approach i but with noninducible clearance producing DHA. These explorations did not improve model fit, as judged by the OFV change and visual diagnostics.

BSV was estimated for all structural parameters except for the *E*_max_ of the autoinduction effect of ARM, due to instability of the model. The addition of BOV on both MTT (ΔOFV = −12.6) and F (ΔOFV = −9.39) yielded significant improvements in the fit. The final model parameter estimates for both ARM and DHA are presented in Table 2, and the VPC of the ARM-DHA model is presented in Fig. 2.

### Covariates.

During the model building, various body size descriptors (i.e., total body weight, ideal body weight [IBW], fat-free mass [FFM], and normal fat mass [NFM]) were implemented allometrically on structural model parameters of LF and ARM-DHA and explored. For both LF and ARM-DHA, none of the applied body size descriptors contributed to model improvement. Considering biological plausibility and previous population PK reports, total body weight, centered to median body weight, was implemented allometrically on clearances (raised to the power of 0.75) and volumes of distribution (raised to the power of 1.0).

Pregnancy has been reported to have significant impact on antimalarial drugs (15, 24). However, in this study, no pregnancy-related covariate effects were found for LF, ARM, or DHA. During the covariate analysis, observed parasitemia density on MTT, EGA on F (described linearly and with a spline), and dosing occasion (OCC) on F (described exponentially) for LF were all selected during the forward selection step (*P* ≤ 0.05; 1 degree of freedom); however, these were not maintained in the backward elimination step (*P* ≤ 0.01).

As for ARM-DHA, none of the covariates tested (time-varying parasitemia density and EGA) had any effect on the PK parameters except for baseline parasitemia density on MTT in the forward step, but again this parameter was not maintained during the backward elimination step (*P* ≤ 0.01). The final PK models for ARM-DHA and LF therefore incorporated only body weight implemented allometrically on clearances and volumes of distribution.

Efforts to simultaneously estimate the PK models for LF and ARM-DHA and explore the correlation between these two drugs' PK parameters, particularly as an effort to assess the correlation in the observed variability in the absorption parameters of both drugs, were not successful and not pursued further because (i) adding off-diagonal correlation elements to explore MTT correlations resulted in an unstable model, and (ii) implementation or correlations in F produced only very minimal and clinically nonsignificant improvements of fit (ΔOFV = −5.54). Moreover, simultaneous modeling substantially increased the computational time (>18-fold increase).

### Model-based simulations of alternative dosing regimens for LF.

**FIG 3:**
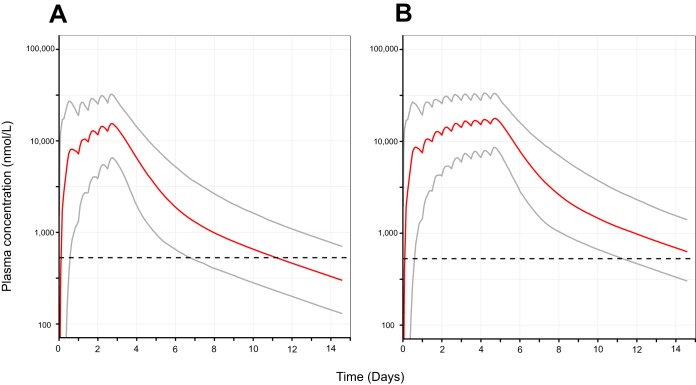
Simulated profiles of lumefantrine plasma concentrations for different dosing regimens. (A) Standard treatment of four tablets of artemether-lumefantrine (one tablet contains 20/120 mg of artemether/lumefantrine) twice a day for 3 days (0, 8, 24, 36, 48, and 60 h); (B) four tablets twice a day for 5 days (0, 8, 24, 36, 48, 60, 72, 84, 96, and 108 h). Solid gray lines represent the 5th and 95th percentiles, while the solid red line represents the mean of the simulated concentration. The dashed lines represent the 280 ng/ml (529 nM) target day 7 LF plasma concentration.

Since day 7 LF concentrations were not available for all patients (samples on exactly day 7 were available for 12/22 patients; samples between days 6 and 10 were available for 16/22 patients), model-based predictions were used to compare PK target attainment of this standard regimen and the alternatively proposed 5-day regimen for pregnant patients (13) (Fig. 3). The simulated median (range) day 7 LF plasma concentration after the standard dosing regimen was 709 (269 to 1,940) ng/ml (corresponding to 1,340 [509 to 3,670] nM), while for the alternative dosing regimen of 5 days this was 2,010 (769 to 4,580) ng/ml (corresponding to 3,801 [1,450 to 8,650] nM).

In terms of PK target attainment, for the standard dosing regimen 11.5% of patients achieved a day 7 concentration below the target concentration, while this was only 0.3% for the alternative dosing regimen.

## DISCUSSION

The shift towards adopting ARM-LF combination therapy in pregnant women primarily in the second and third trimesters has been widely accepted (4). Several previous studies on LF and ARM-DHA exposure in pregnant women have shown that this is a critical determinant of malaria treatment outcome, particularly preventing recrudescence (7).

For our study, the final LF population PK model with an absorption model consisting of five transit-compartment followed by two disposition compartments adequately described the observed LF plasma concentrations following a standard 3-day regimen in our population of second- and third-trimester pregnant patients from Rwanda. The data were sparse at the extended sampling duration; however, the applied informative prior approach enabled the implementation of a peripheral compartment since LF had been described to exhibit a multicompartmental disposition (15).

Most of the LF PK parameter estimates in our study were in good agreement with those previously described, particularly in Uganda and Tanzania (15, 25). The large variability in the data was captured through the large estimated variabilities (BOV, BSV, and residual error), particularly in the parameters MTT and F. This might be the result of food intake together with LF administration (9, 26, 27) or disease-induced changes (parasite density changes from infection to recovery). LF is a lipophilic compound for which a fatty meal is needed to enhance its F (27). In this study, patients were given a standardized glass of milk and/or a small cake with supervised administration of artemether-lumefantrine (Coartem). Though food was standardized, the variability in food intake due to ability of food consumption could have affected bioavailability (27).

Good clinical and parasitological outcome was observed in this study; i.e., all patients had a microscopic parasite density below the observable limit by day 3 after the first dose, and only a very low recrudescence rate (4.55%; 1/22 patients) was observed in this study (data not shown). The simulations performed with the final LF PK model indicated that adequate exposure in pregnant women was achieved with current standard dosing; the simulated median day 7 LF concentration in our population was 709 ng/ml, which was somewhat lower than reported from Tanzania (908 ng/ml) (25) but almost twice as high as the values reported from Uganda (414 ng/ml) and the Thailand-Myanmar border (431 ng/ml) (15, 25, 28). In addition, the value in this study was higher than the reported day 7 LF concentration in nonpregnant patients from the northwestern border of Thailand (528 ng/ml) and from Laos (470 ng/ml) (12, 29). Moreover, according to our simulations the percentage of patients who failed to attain the day 7 target LF concentration (280 ng/ml) was much lower (11.5%) than reported in previous studies for pregnant women using this dose regimen (i.e., 35% and 32% in Thailand and Uganda, respectively) and was almost similar to that in a study from Tanzania (9.0%) (6, 15, 25). This would indicate that the LF exposure was not compromised in this pregnant cohort from East Africa compared to cohorts in Southeast Asia, which is consistent with other findings from the East African region, i.e., in Uganda and Tanzania (25, 28).

Nevertheless, increasing the duration of the standard ARM-LF regimen from 3 days to 5 days has previously been suggested to achieve higher day 7 LF concentrations in pregnant women than does a 100% dose increase over 3 days (13). Simulations using the 5-day regimen in the present study also exhibited day 7 LF concentrations 2.8-fold higher than for the standard 3-day regimen. The extended 5-day regimen would also cover 1 additional Plasmodium life cycle; however, treatment adherence and increased cost could be challenges limiting implementation. The relatively high PK target attainment and the good clinical outcome in the current pregnant cohort from Rwanda using the conventional 3-day ART-LF regimen combined with the practical limitations associated with the 5-day regimen might question whether there is need to further extend the current ARM-LF treatment in pregnant women in East Africa (13, 25).

A simple one-compartment model with rapid absorption in combination with a CYP3A4 enzyme turnover model to characterize the autoinduction of ARM clearance was used to describe both the parent ARM and metabolite DHA PK (21, 25). ARM exhibited fast but erratic absorption, which was evident through high variability estimates for both MTT and F parameters (21, 25). There are studies that related the observed time dependency of ARM clearance to the gradually improving health status of malaria patients (7). However, the observed decreasing peak concentration of ARM in combination with the increasing peak concentration of DHA during the treatment period would indicate enzymatic autoinduction rather than disease-related changes (17, 20). ARM is metabolized *in vitro* to its active metabolite DHA by enzymes CYP1A2, CYP2B6, CYP3A4, and CYP3A5 (30); contribution by CYP2C19 and CYP2D6 has not been observed in healthy subjects (18). CYP3A4 plays the largest role in ARM metabolism *in vitro* (31, 32). DHA is eliminated subsequently by glucuronidation, most likely mediated by UGT1A9 and UGT2B7 (31, 33). Autoinduction of hepatic clearance can be modeled in many ways, but the enzyme turnover model implemented for ARM in this study is in line with the assumption that the ARM concentration affects only ARM clearance and not the clearance of DHA, i.e., inhibition of metabolite formation (34). With a modest increase in the induction enzyme activity over time, the enzymatic half-life was estimated at 30.6 h (95% CI, 7.59 to 41.9), which is slightly lower than previously estimated *in vivo* CYP3A4 half-lives (35–37). For CYP3A4 a wide range of half-lives has been reported *in vitro* and *in vivo*, between 2 and 158 h (35–38). The influence of coinduction of other CYP enzymes for which ARM is a substrate, such as CYP3A5, could be the reason for the relatively low estimated enzyme half-life (32).

A more mechanistic allometric implementation of alternative body size descriptors, such as FFM, IBW, and NFM, on clearances and volumes of distribution was explored. Our attempt to distinguish the influence of pregnancy on fat and fat-free components of the body mass and subsequently on PK properties of these drugs (primarily LF) proved inconclusive. The narrow distribution of patients' body weight, lack of nonpregnancy data for comparison, and relatively small sample size could be the reason for this. In general, suitable body size descriptors to describe the dynamic changes in maternal body weight and PK property changes associated with this have not been explored extensively (39).

An effect of EGA as a covariate on the distribution volumes has been reported previously for LF (13), which could be justified by the prominent physiological changes in pregnant women between trimesters two and three (especially those in late pregnancy). In this study, EGA was not identified as a significant covariate for LF and ARM-DHA, probably since the range of EGA was quite limited (16 to 39 weeks), our data set was relatively sparse, and the study lacked nonpregnant controls. Pooled data analyses to study pregnancy effects in more detail could be considered. There have been previous reports failing to identify differences between trimesters two and three of pregnancy; e.g., for ARM-DHA no difference in CYP3A4 activity between the second and third trimesters could be observed (24, 40, 41). The magnitude of enzyme autoinduction of ARM would be particularly interesting to explore in pregnant versus nonpregnant patients (postpartum), since significant increases of CYP3A4 enzymes during pregnancy have been reported (7, 40, 42).

Parasite density over time was evaluated as a covariate since it has been reported previously to be a significant covariate on F and MTT for artesunate and artemisinin in malaria patients, constituting a disease severity effect on the absorption of these drugs (43, 64). However, it was not found to be significant in our study, either for LF or for ARM-DHA. Patients had a relatively narrow baseline parasite density and were cleared of parasitemia within 2 to 3 days of treatment, indicating good initial parasitological response.

ARM-LF is administered in a fixed-dose combination. LF has been previously reported to affect ARM PK, where it increased the absorption rate of ARM (17). Moreover, since the absorption of ARM-DHA is highly variable and erratic, we tried to decrease unexplained variability by implementing a correlation between absorption parameters of LF and ARM-DHA. This simultaneous modeling approach was unfortunately not successful, and due to the instability of the combined model, the two drugs were eventually modeled separately. Since the mean absorption times differed substantially between the two drugs (45 min versus 4 h), different physiological aspects and dosing-related conditions might indeed dictate variability in these parameters.

In conclusion, population PK modeling allowed successful characterization of the PK properties of LF, ARM, and DHA, including autoinduction of ARM clearance, in pregnant women with uncomplicated Plasmodium falciparum malaria in Rwanda. The use of a population approach enabled the investigation of the effect of variables such as various body size descriptors and EGA on these simultaneously administered antimalarial drugs. Though exposure of LF was found not to be compromised in pregnant women in this East African study population by evaluation of the PK target day 7 LF plasma concentrations, larger, better-structured studies are needed to be more conclusive about ARM-LF dose extension during pregnancy, also considering the recent emergence of artemisinin resistance.

## MATERIALS AND METHODS

### Study design.

This PK study was nested within a pharmacovigilance study reported elsewhere on the use of ACT in pregnancy during acute malaria (44). The study was conducted in the obstetrics and gynecology ward of Rwamagana district hospital in Rwanda. Rwamagana is in the eastern province of Rwanda, with mesoendemic malaria transmission intensity, and the study took place from June 2007 to July 2009. The study was approved by the Rwanda National Ethics Committee as study number IRB 00001497.

Briefly, eligible pregnant women older than 18 years with uncomplicated Plasmodium falciparum malaria, confirmed by light microscopy, were recruited once signed informed consent was obtained. Pregnancy was confirmed by a human chorionic gonadotropin pregnancy urine test, and gestational age was estimated by ultrasound. Enrolled patients were prescribed 4 tablets of a fixed oral combination of ARM and LF twice daily under supervision for 3 days (at 0 h [initial dose] and 8, 24, 36, 48, and 60 h) with a glass of milk and/or a small cake (a fatty meal) to enhance absorption. Novartis, Basel, Switzerland, provided the tablets, and each tablet contained 20 mg of ARM and 120 mg of LF.

### Blood sampling and assay.

Just before first drug administration (time < 0 h), blood was collected for biochemistry and the remaining plasma was used as the first, pre-drug administration (time < 0 h) PK sample. Thereafter, PK samples were collected through venipuncture. Blood samples were collected at 2 and 4 h after each dose and just before doses 2, 3, 4, 5, and 6 to measure trough concentrations. Additional samples were taken at 0.25 h after doses 1 and 2. Samples were collected at 6, 8, and 12 h after the last dose, in addition to a scheduled sample at day 7 after initiation of treatment. Patients were admitted until the last sample was taken and parasite clearance achieved. A total of 4 ml of blood per sampling time was collected in glass lithium-heparin vacuum tubes with gel. The sodium heparin tubes were at room temperature (18°C to 25°C) prior to use. Samples were centrifuged without delay and the plasma was separated and frozen at −70°C. Samples were shipped on dry ice to the Department of Clinical Pharmacology, Mahidol-Oxford Tropical Medicine Research Unit, Bangkok, Thailand, for drug measurements. The laboratory is accredited according to ISO15189 and ISO15190. Drug concentrations of ARM and DHA were measured in plasma using high-performance liquid chromatography coupled with tandem mass spectrometry (45); meanwhile, LF was measured using automated solid-phase extraction (46). The LLOQ were 1.43 ng/ml for both ARM (4.79 nM) and DHA (5.03 nM) and 24.86 ng/ml (47 nM) for LF. Samples that showed signs of extensive hemolysis were excluded from analysis. Quality control samples of LF, ARM, and DHA at three levels (low, middle, and high) were analyzed within each batch of clinical samples to ensure precision and accuracy during routine clinical drug measurements. The coefficients of variation for all analytes were lower than 5% for all quality control samples, which is well below the required precision of ±15% according to U.S. FDA regulatory guidelines (47, 48). Parasitemia density assessment was conducted by microscopy using Field's method, i.e., thin or thick blood film counts of asexual parasites and gametocytes every 8 h (±1 h) following the first dose administration until 72 h postdose (48, 49). After discharge from the hospital, samples for thick and thin blood films were collected on outpatient basis on days 7, 14, 21, 28, 35, 42, 49, and 56 or on any other day when clinically indicated.

### Population pharmacokinetic and pharmacodynamic modeling.

Estimation and simulation were performed using nonlinear mixed-effects modeling in the software NONMEM 7.3 (ICON Development Solutions, Ellicott City, MD) (50). Postprocessing, diagnostics plots and automation were performed using Perl-speaks-NONMEM (PsN, 4.5.3) (51), Xpose (4.5.3) (52), Pirana (2.9.2) (53), and R (3.2.4) (54).

### Population pharmacokinetics.

The molar units of LF, ARM, and DHA concentration were transformed to their natural logarithms for this modeling analysis. All the BLLOQ data for ARM and DHA were included and explored with the application of the likelihood-based M3 method for censored observations using the Laplacian estimation method (55, 56). Conversely, LF BLLOQ data were not included, as they made up <10% of the total data, and the FOCEI (first-order conditional estimation with interaction) estimation method was used. The unexplained residual error was estimated using an additive error model on the logarithmic scale for all drugs, which equates to an exponential error model on an arithmetic scale. In the case of ARM and DHA, a separate additive error model was used for each analyte. Different structural absorption (first-order, first-order with transit compartment, and sequential absorption) and distribution (one-, two-, and three-compartment) models were explored for all drugs.

Because of LF data sparseness, particularly during the elimination phase, informative priors based on a previous study (15) were applied to all parameter estimates (57). The chosen prior model explored PK properties of LF in pregnant and nonpregnant women with uncomplicated Plasmodium falciparum malaria in Uganda. The final model had pregnancy retained as a significant covariate on intercompartmental clearance. Hence, the frequentist prior estimation for LF's Q/F was recalculated to represent estimation for pregnant women in this study. The typical relative F was implemented as a fixed parameter for the parent analyte, i.e., LF and ARM (100% relative bioavailability). The stochastic model implemented consisted of BSV modeled as shown below (equation 2), BOV, and residual variability. Individual parameters for both drugs were modeled as lognormally distributed around the population estimate, except for F of LF. Box-Cox transformation (58) was explored for the distribution of BSV on F as shown below (equation 3) to assess formally the assumption that PK parameters are lognormally distributed.

(2) $$P_{i}=P_{\text{pop}}\times e^{\eta }$$

(3) $$P_{i}=P_{\text{pop}}\times e^{\eta^{\frac{\lambda \;-\;1}{\lambda }}}$$

where *P_i_* represents the individual parameter estimate, *P*_pop_ represents the typical parameter estimate for the population, η represents the BSV, and λ represents the estimated Box-Cox transformation factor.

ARM is known to exhibit an autoinduction of its own clearance (17). Enzyme kinetics was included in the ARM-DHA PK model, and an enzyme turnover model used previously by Hassan et al. and Smythe et al. was also adapted in this study (37, 59). ARM and DHA, expressed as molar concentrations, were characterized simultaneously assuming complete and irreversible *in vivo* conversion of ARM into DHA.

The dynamics of the enzyme compartment over time was expressed as shown below (equation 4); *A*_ENZ_ is the amount of enzyme in the enzyme compartment, *K*_ENZ_ is the first-order degradation rate constant of the enzyme, and EFF is the link between ARM concentration and its enzyme pool through increase in enzyme production rate. Linear and nonlinear relationships (*E*_max_ model) describing the effect of ARM concentrations on the induction of its own clearance were explored.

(4) $$\frac{{dA}_{\text{ENZ}}}{dt}=K_{\text{ENZ}}\times (1+\text{EFF})-K_{\text{ENZ}}\times A_{\text{ENZ}}$$

The enzyme concentration was initialized at 1 in order to normalize it to unity at baseline; i.e., the zero-order production rate of the enzyme was set to *K*_ENZ_. This (enzyme) then modulates the preinduced ARM clearance (equation 5).

(5) $${\left({\text{CL}}_{\text{ARM}}/F\right)}_{\text{induced}}={\left({\text{CL}}_{\text{ARM}}/F\right)}_{\text{preinduced}}\times A_{\text{ENZ}}$$

The model was parameterized in such way that the enzyme half-life (*t*_1/2ENZ_) was estimated as shown below (equation 6).

(6) $$K_{\text{ENZ}}=\frac{\text{ln}2}{t_{1/2\text{ENZ}}}$$

The body size descriptor covariates (total body weight, IBW, FFM, and NFM), EGA, observed baseline parasitemia density, observed time-varying parasitemia density, temperature, and dosing occasion (OCC; i.e., each dose given was considered single dosing occasion) were considered for exploration of covariate analysis for LF, ARM, and DHA based on biological plausibility and previous findings.

For body size descriptors, different covariate implementations were explored: allometric scaling using total body weight, allometric scaling using IBW (60) (equation 7), allometric scaling using FFM (equation 8), and allometric scaling using NFM (equation 9), with *Ffat* representing the contribution of fat mass normalized to the FFM estimated for (i) CL/F, Q/F, volume of distribution of central compartment (*V_c_*)/F, and *V_p_*/F for LF and (ii) apparent clearance and volume of distribution of central compartment for ARM (CL_ARM_/F and *V*_2_/F) and DHA (CL_DHA_/F and *V*_3_/F) (61).

(7) IBW=45.4+0.89×(height in cm−152.4)

(8) $$\text{FFM}=\frac{{\text{WHS}}_{\text{max}}+\text{height in}\;m^{2}\times \text{weight in kg}}{{\text{WHS}}_{50}+\text{height in}\;m^{2}\times \text{weight in kg}}$$

(9) $$\text{NFM}=\text{FFM}+{Ffat}_{\frac{\text{CL}}{F}\text{or}\frac{V}{F}}$$

where WHS_max_ is 37.99 kg/m^2^ and WHS_50_ is 35.98 kg/m^2^, which represent the maximal and half-maximal weight-for-height standards, respectively.

All size descriptors were scaled to their respective medians (i.e., total body weight [59 kg], FFM_median_, IBW_median_, and NFM_median_) on PK parameters using allometric power exponents of 0.75 for clearances (CL/F, Q/F, CL_ARM_/F, and CL_DHA_/F) and 1 for volumes of distribution (*V_c_*/F, *V_p_*/F, *V*_ARM_/F, and *V*_DHA_/F).

An example implementation for body descriptor FFM on CL/F is shown below (equation 10):

(10) $$\text{CL}/F_{i}=\text{CL}/F_{\text{pop}}\times {\left(\frac{{\text{FFM}}_{i}}{{\text{FFM}}_{\text{median}}}\right)}^{0.75}$$

Later, all chosen covariates were explored with body weight maintained on clearances and distribution volumes using a standard allometric function when evaluating linear, exponential, and power relationships for the other covariates, which were normalized to their median values in the population.

Stepwise covariate modeling was applied for all continuous covariates using *P* values of 0.05 (ΔOFV > 3.84; 1 degree of freedom) in the forward step and 0.01 (ΔOFV > 6.63) in the backward step (62). EGA was additionally explored as a categorical covariate (trimester 2 versus 3) using a forward inclusion cutoff ΔOFV of >5.99 (2 degrees of freedom).

The final separate LF and ARM-DHA PK models were subsequently evaluated as a combined model in a simultaneous fit, to explore correlations between the PK parameters of both drugs, in particular bioavailability and absorption rate. Parameter correlations were explored using the variance-covariance matrix.

### Model-based simulations of alternative dosing regimens for LF.

Monte Carlo simulations (*n* = 2,200) were performed using the final LF population PK model for up to 15 days after the first dose to assess and compare PK target attainment after a standard dosing regimen (80 mg of ARM/480 mg of LF twice daily for 3 days) and an extended alternative dosing regimen (80 mg of ARM/480 mg of LF twice daily for 5 days) in this particular population (13). The predicted median and 5th and 95th percentiles for LF concentration were extracted from their simulated distributions to assess descriptively the day 7 concentration target attainment (280 ng/ml).

### Model evaluation.

Model selection was guided by physiological plausibility, plausible parameter estimates, precision of parameters, visual diagnostics, and OFV, computed by NONMEM as minus twice the log likelihood where a drop in OFV of 3.84 or more was considered a significant (*P* = 0.05) improvement between two hierarchical models after inclusion of one additional parameter (1 degree of freedom). VPCs were performed (1,000 simulations) to evaluate the predictive performance of developed models. The 5th, 50th, and 95th percentiles of obtained data for the respective PK model were overlaid with the model-predicted 95% confidence intervals of the same percentiles. Diagnostic plots were used to evaluate the overall goodness of fit by plotting log-transformed observed drug concentrations against the population fitted and the individually fitted log-transformed concentrations. The reliability of individual parameter estimates and goodness-of-fit plots were also assessed through eta and epsilon shrinkages. Estimates of parameter imprecision were obtained from a recently developed procedure, sampling importance resampling (SIR) (63).
